# Availability and affordability of essential medicines for non-communicable disease management in primary healthcare: Evidence from three municipalities in Ghana

**DOI:** 10.1371/journal.pone.0346140

**Published:** 2026-04-02

**Authors:** Mary Yeboah, Richard Abeiku Bonney, Loretta Adu-Boahemaa Antwi, Pius Amponsah Anane, Obed Kwabena Offe Amponsah, Peter Agyei-Baffour

**Affiliations:** 1 School of Public Health, Kwame Nkrumah University of Science and Technology, Kumasi, Ghana; 2 Department of Health Care Management, Technische Universität Berlin, Berlin, Germany; 3 Department of Pharmacy Practice, Kwame Nkrumah University of Science and Technology, Kumasi, Ghana; University of Westminster - Regent Street Campus: University of Westminster, UNITED KINGDOM OF GREAT BRITAIN AND NORTHERN IRELAND

## Abstract

**Background:**

Non-communicable diseases (NCDs) cause 74% of global deaths, disproportionately affecting LMICs like Ghana. Chronic treatment remains hindered by medicine shortages and high costs, consuming over 50% of the minimum wage incomes. Despite Ghana’s NCD policies, supply chain gaps and price inflation persist. This study assesses access to medicine, operationally defined as availability and affordability, in three municipalities to inform reforms for Universal Health Coverage and achieve SDG 3.4 targets.

**Methods:**

This cross-sectional mixed study assessed the availability and affordability of NCD medicines in three municipalities in Ghana using WHO/HAI methods. Data on 62 medicines were collected from nine health facilities, supplemented by interviews with pharmacy managers.

**Results:**

This study assessed access to NCD medicines across three municipalities in Ghana, revealing stark disparities. Availability varied significantly by location (Oforikrom 70% vs. Juaben 48.6%, p < 0.001) and facility type (government 67.2% vs. private 54.6%, p = 0.042). Insulin was least affordable (3.94–8.74 days’ wages), with 10-fold metformin price differences between municipalities. Private facilities charged 2–5 × more than the government for chronic medications. Logistic regression analysis showed that patients in private facilities were significantly more likely to encounter unaffordable NCD medicines compared to those in public facilities (AOR = 3.85, p = 0.001). Supply chain delays (2–3 weeks) and National Health Insurance Scheme (NHIS) reimbursement delays exacerbated gaps.

**Conclusion:**

This study highlights inequities in access to NCD medicines in Ghana, with stark gaps in availability and affordability, especially in underserved areas. Findings reveal high costs, stock-outs, and geographic disparities as key factors, underscoring the need for supply chain reforms, price regulation, and municipal oversight to advance equitable, sustainable, universal health coverage.

## Background

Non-communicable diseases (NCDs) represent a global public health challenge, accounting for 74% of all deaths worldwide [1], with 73% of these deaths occurring in low- and middle-income countries (LMICs) [2]. Unlike communicable diseases, NCDs are characterized by their chronicity, multifactorial etiology, and strong association with modifiable behavioral and metabolic risk factors, including physical inactivity, unhealthy diets, tobacco use, harmful alcohol consumption, and air pollution [3,4].

Non-communicable diseases are a growing public health concern in Sub-Saharan Africa (SSA), with wide variation in prevalence across the region. Reported rates range from 0.07–0.3% for stroke, 0–16% for diabetes, 6–48% for hypertension, 0.4–43% for obesity, and 0.4–71% for smoking [5]. Globally, NCDs account for 71% of all deaths, primarily due to cardiovascular diseases, cancers, chronic respiratory diseases, and diabetes [6]. In SSA, NCDs such as hypertension and diabetes contribute substantially to morbidity and mortality and affect younger populations compared to high-income countries, placing additional strain on already burdened health systems [7].

In Ghana, NCDs contribute to 43% of total mortality, with rising prevalence linked to urbanization, demographic shifts, and inadequate health system preparedness [8,9]. The economic and healthcare burden is exacerbated by limited access to essential medicines, a cornerstone of NCD management. Chronic conditions like hypertension and diabetes require lifelong treatment, yet stock-outs, affordability barriers, and supply chain inefficiencies persistently undermine therapeutic adherence and outcomes [10,11]. For instance, a study across 36 LMICs revealed that public-sector availability of NCD medicines averaged only 36%, compared to 55% in the private sector; often at prohibitive costs [12].

Ghana’s Ministry of Health (MoH) launched its NCD Policy (2012) with support from WHO and the World Bank, aligning with the WHO Global Action Plan (GAP) 2013–2020 to strengthen health systems for NCD prevention and control [13]. However, implementation gaps persist. The WHO defines access as the availability of medicines within one hour’s walk of households, coupled with affordability, measured against the daily wage of the lowest-paid government worker [14]. In Ghana, however, economic instability, retail markups (up to 45%), and foreign exchange volatility inflate medicine prices, rendering them unaffordable for many [15,16]. For example, the lifetime cost of hypertension treatment in Ghana ranges from GHS 570,239 to 1.202 million (USD 78,115–164,723), accounting for over 50% of a minimum-wage earner’s income [11,17]. Such financial toxicity forces households into catastrophic health expenditures, perpetuating cycles of poverty [18].

Studies in Ghana and comparable LMICs (e.g., Kenya, Uganda, Nigeria) highlight systemic bottlenecks in access to NCD medicines. A critical issue is the frequent stock-outs of essential medicines, with over 30% routinely unavailable in public health facilities, undermining treatment continuity [10]. Geographic disparities further exacerbate inequities, as rural-urban divides limit access for underserved populations [19]. Weak supply chains, characterized by fragmented procurement and distribution systems, disrupt the reliable availability of medicines [20]. Compounding these challenges are regulatory gaps, including inconsistent pricing policies and insufficient government subsidies, which deepen affordability crises for patients [11].

Despite Ghana’s progressive National Medicines Policy (2017), empirical data on access to NCD medicines in the Ashanti Region, particularly in the Municipalities of Oforikrom, Ejisu, and Juaben, remain scarce. This region faces unique challenges due to its mixed urban and peri-urban demographics. High NCD prevalence coexists with fragmented primary healthcare networks, straining already limited resources [18]. While few studies directly assess the affordability of medicines here, proxy data indicate that retail markups often exceed 50% for antihypertensives and antidiabetics, placing these therapies out of reach for many [21].

Achieving Universal Health Coverage (UHC) and SDG 3.4, which targets a 33% reduction in premature NCD mortality by 2030, hinges on equitable access to medicines [22]. This study addresses a critical evidence gap by evaluating the availability and affordability of essential NCD medicines in three (3) municipalities in the Ashanti region. The findings will inform policy reforms to advance sustainable medicine financing, such as expanding NHIS coverage and strengthening supply chains through context-specific interventions. Additionally, the study provides actionable insights to mitigate financial hardship for patients through equitable pricing mechanisms, aligning with Ghana’s commitment to health equity.

## Methods

### Study design

This study employed an analytical cross-sectional design, using the World Health Organization and Health Action International (WHO/HAI) standardized assessment methodology [23] to assess the availability and affordability of essential NCD medicines in primary healthcare facilities across three municipalities in Ghana’s Ashanti Region (Oforikrom, Ejisu, and Juaben). The design enabled a snapshot evaluation of medicine supply and pricing dynamics, aligning with the study’s objectives to inform policy reforms. The WHO/HAI methodology was selected for its robustness in facilitating international comparisons of medicine prices and availability. Data were collected on 62 essential NCD medicines from the WHO PEN Tool list, excluding cancer therapies (managed at tertiary levels) and adjusting for local guidelines (e.g., replacing Senna with Lactulose). A mixed-methods approach integrated quantitative data on medicine prices, availability, and affordability metrics with qualitative data from key informant interviews with pharmacy managers to contextualize supply chain barriers.

### Sampling and participants

For facility selection, nine medicine outlets were purposively sampled across the three municipalities, stratified by facility type: three government hospitals (anchor facilities per WHO/HAI criteria), three mission/Christian Health Association of Ghana (CHAG) hospitals, and three private hospitals. Facilities were selected based on capacity for NCD management (presence of medical officers/physician assistants). Nine pharmacy managers were purposively selected for interviews to gain insights into supply chain challenges; inclusion criteria required at least 6 months of work in the NCD department.

The decision to select an equal number of facilities (three per municipality) was informed by methodological considerations rather than the actual number of facilities in each Municipality. The purposive sampling strategy aimed to ensure comparability across municipalities while maintaining representation from the three key facility types: government, CHAG/mission, and private hospitals.

Although the municipalities may not have the same total number of facilities, selecting an equal number from each allowed us to control for variation in facility type and capacity for NCD management across districts. This approach also aligns with the WHO/HAI methodology, which emphasizes selecting anchor facilities with a consistent sampling frame, especially when the objective is to compare availability, pricing, and service readiness across regions.

A total of 549 patients were surveyed across the selected health facilities. The sample size was determined in line with the WHO/HAI standardized methodology for affordability assessments, which recommends surveying a sufficient number of patients accessing medicines for chronic conditions to ensure stable estimates of affordability across facility types [23,24]. Given the absence of prior facility-level estimates for NCD medicine affordability in the study area, the final sample size was guided by feasibility considerations, patient flow at outpatient pharmacies during the data collection period, and the need to ensure representation across municipalities and facility types.

Patients were recruited using a systematic consecutive sampling approach at outpatient pharmacy units in each selected facility. All adult patients (≥18 years) diagnosed with at least one non-communicable disease and prescribed one or more medicines included in the WHO PEN list were eligible for inclusion. Patients were approached after completing their pharmacy encounter and invited to participate until the facility-specific sample quota was reached. Patients who were critically ill or unable to provide informed consent were excluded.

To ensure proportional representation, the total sample was distributed across facilities based on average outpatient pharmacy attendance during the study period. Consequently, patient recruitment per facility ranged from approximately 60–63 patients (see Table 1).

**Table 1:** Sample distribution across municipalities and facility types.

Facility type/Municipality	Oforikrom (n = 186)	Ejisu (n = 182)	Juaben (n = 181)	Total
**Public**	63	60	60	183
**Missionary**	61	61	61	183
**Private**	62	61	60	183
**Total**	186	182	181	**549**

### Data collection procedure

Medicine availability and pricing data were collected by trained research assistants who physically verified stock and recorded prices of the lowest-priced generics using WHO/HAI forms. Data were collected at the various health facilities involved in the study, with double-entry validation to minimize errors. For the affordability assessment, costs were calculated as the price of a 30-day treatment course relative to the prevailing minimum daily wage (GHS14.88; USD1.65) of Ghana’s lowest-paid unskilled government worker (2023 baseline) [25]. Semi-structured qualitative interviews explored barriers to access to medicine, including procurement delays and pricing policies. All data collection tools were refined following pilot testing at Effiduase District Hospital (Sekyere-East District), with minor revisions made to clarify dosage units, improve item sequencing, and capture intermittent stockouts and brand substitutions, while retaining the core WHO/HAI indicators. The data collection started from September 2, 2023, to June 13, 2024.

### Study setting

The study was conducted in three municipalities, Oforikrom, Ejisu, and Juaben, within Ghana’s Ashanti Region, a populous area with a mix of urban and peri-urban communities. Healthcare services in the region are delivered through government, mission/CHAG, and private facilities, all providing outpatient management of NCDs. Selected facilities included government hospitals serving as anchor sites, complemented by mission/CHAG and private hospitals to represent the main types of NCD care providers in the municipalities. The setting reflects routine primary and secondary healthcare contexts where patients access essential NCD medicines, providing a representative basis for assessing medicine availability and affordability.

### Data management

Data were collected using standardized WHO/HAI data collection tools and managed using Microsoft Excel (Microsoft Corporation, Redmond, WA, USA; version 2019) and IBM SPSS Statistics for Windows (version 25.0; IBM Corp., Armonk, NY, USA) for analysis. Quantitative data on medicine availability, prices, and affordability were double-checked for completeness and consistency at the point of entry. Unique identification codes were assigned to each facility and medicine to ensure accurate tracking and prevent duplication. Qualitative interview notes were anonymized and securely stored. All datasets were password-protected and accessible only to the research team. Data cleaning procedures included range checks, validation against source documents, and resolution of discrepancies through cross-verification with field records to ensure data quality and integrity.

### Data analysis

**Fig 1:**
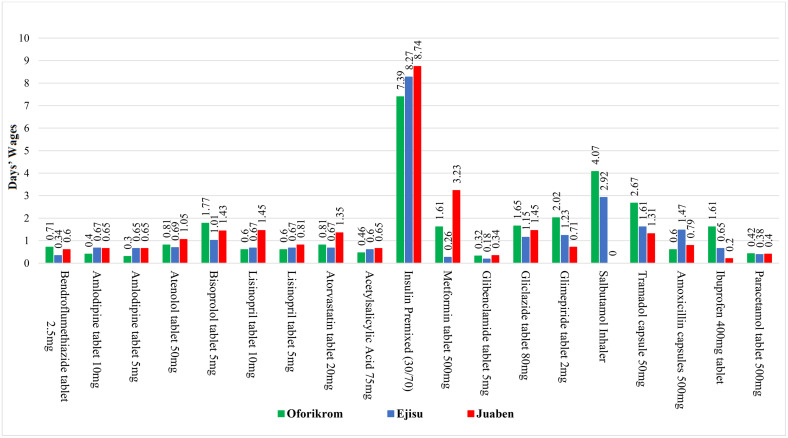
Affordability of selected NCD medicines across various municipalities for non-insured patients.

**Fig 2:**
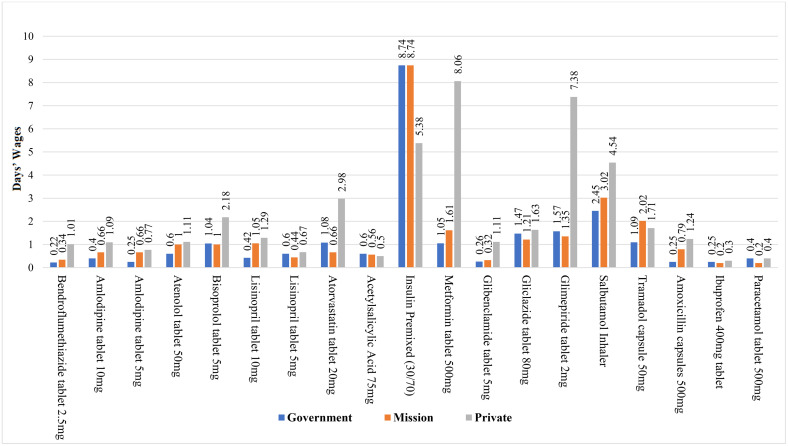
Affordability of selected NCD medicines across various facility types for non-insured patients.

**Fig 3:**
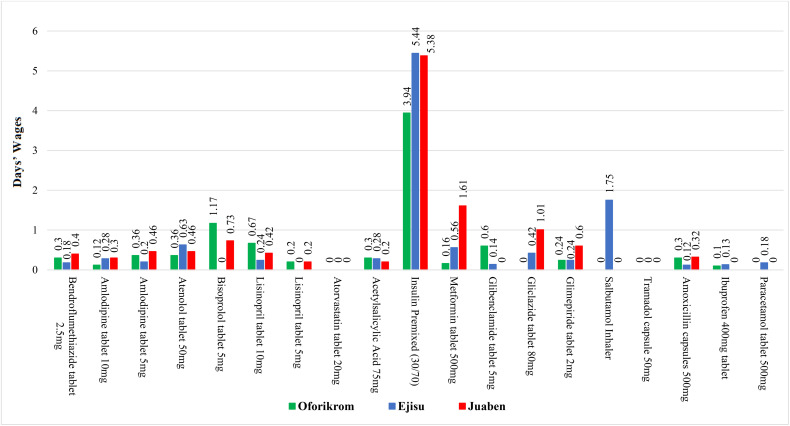
Affordability of selected NCD medicines with added-on cost for insured patients across municipalities.

**Fig 4:**
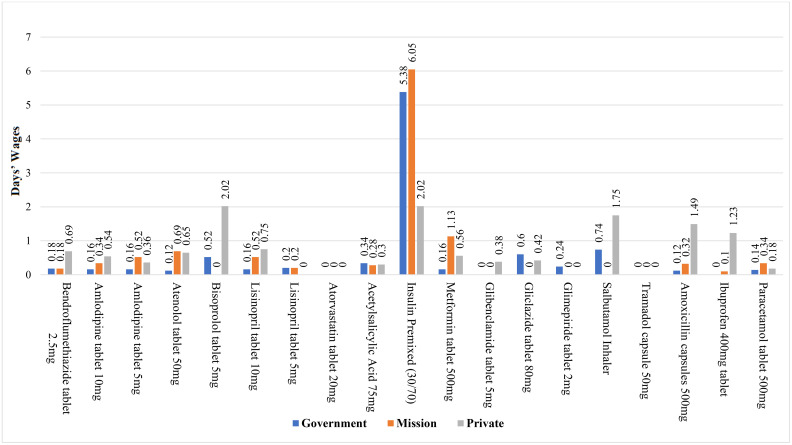
Affordability of selected NCD medicines with added-on cost for insured patients across facility types.

Quantitative data on medicine availability, prices, and affordability were entered and analyzed using IBM SPSS Statistics for Windows (version 25.0; IBM Corp., Armonk, NY, USA) and Microsoft Excel (Microsoft Corporation, Redmond, WA, USA; version 2019). Availability was categorized as very low (<30%), low (30–49%), relatively high (50–80%), or high (>80%), consistent with the thresholds proposed by Dabare et al. [20]. Figs 1–4 were generated from the facility-level availability and price datasets and constructed using descriptive statistics in Excel and SPSS to illustrate variations in medicine availability, median price ratios, and affordability across facility types and municipalities. Logistic regression analysis was performed to examine the association between facility type and medicine affordability, adjusting for municipality. Statistical significance was assessed at a two-sided p-value < 0.05. Affordability was measured as the number of days of wages required to purchase treatment, and chi-square tests were used to compare affordability across municipalities and facility types. Median Price Ratios (MPRs) benchmark local prices against international reference prices; MPR ≤ 1.5 is considered affordable, and MPR > 2 is considered overpriced (*see* S1 File).

Thematic analysis was conducted on key informant interviews with pharmacy managers at the selected health facilities. Interviews were conducted on-site in private settings, lasted approximately 30–40 minutes, and were audio-recorded with participants’ consent. Recordings were transcribed verbatim, and transcripts were manually coded using an inductive thematic approach. Codes were iteratively reviewed and grouped into themes reflecting essential medicine supply chain challenges, including stockouts, procurement bottlenecks, and distribution delays.

### Reliability and validity

Pilot testing and inter-researcher consistency checks ensured tool reliability. Triangulation of quantitative and qualitative data enhanced the validity of the findings by providing multiple perspectives on access to medical care. The study maintained rigorous quality control measures throughout data collection and analysis to ensure robust results. These included interviewer training and standardization before data collection, use of pre-tested and validated WHO/HAI data collection instruments, range checks and consistency checks during data cleaning in Microsoft Excel, and independent verification of a random sample of entered data against original field forms to assess accuracy.

### Ethical approval and consent to participate

Approval was obtained from the Committee on Human Research, Publications and Ethics (CHPRE) at the Kwame Nkrumah University of Science and Technology with reference number CHRPE/AP/771/23. Administrative approvals were sought and obtained from all municipal health authorities and the leadership of participating hospitals. This study adhered to the Helsinki Declaration. All participants provided written consent, and data were anonymized. Facility identities were coded to ensure confidentiality throughout the research process.

## Results

### Demographics of municipalities and health facilities

To assess patients’ experiences with the availability and affordability of medicines, 549 patients were surveyed across the selected facilities (see Table 2). Sociodemographic characteristics of interview participants are presented in Table 3 below.

**Table 2:** Socio-demographic characteristics of patients surveyed across selected health facilities.

Characteristic	Category	n = 549	Percentage (%)
**Age group (years)**	18-29	24	4.4
	30-39	68	12.4
	40-49	95	17.3
	50-59	178	32.4
	≥60	184	33.5
**Sex**	Male	212	38.6
	Female	337	61.4
**Educational level**	No formal education	52	9.5
	Primary	250	45.5
	Secondary	132	24.0
	Tertiary	115	21.0
**Employment status**	Employed	105	19.1
	Self-employed	308	56.1
	Unemployed	136	24.8

**Table 3:** Sociodemographic characteristics of interview participants.

S/N	Age	Gender	Educational level	Profession	Years in service mean = 6.4 years
P01	22	Female	Diploma	Community Health Worker	1
P02	28	Male	Doctor of Pharmacy	Pharmacist	4
P03	44	Male	Bachelor	Pharmacist	13
P04	32	Male	Bachelor	Store Manager	3
P05	55	Male	Bachelor	Pharmacist	14
P06	29	Female	Bachelor	Pharmacist	3
P07	27	Female	Doctor of Pharmacy	Pharmacist	3
P08	42	Female	Doctor of Pharmacy	Pharmacist	1
P09	54	Female	Doctor of Pharmacy	Pharmacist	16

### Availability of NCD medicines

A total of 62 essential medicines for NCDs were surveyed (*see* S2 File). The study reveals striking disparities in the availability of essential medicines across municipalities and facility types, with concerning implications for NCD management. Cardiovascular drugs show a 28-percentage-point availability gap between the best-performing Oforikrom (67%) and the worst-performing Juaben (39%), while critical medications like atenolol 25 mg remain entirely unavailable. Diabetes medicines present an inverse pattern, with Ejisu leading (76%) and government facilities outperforming private providers by 29 percentage points (86% vs 57%), though insulin access remains problematic. Asthma treatments demonstrate the most severe shortages (20–53% availability), compounded by the complete absence of beclomethasone inhalers nationwide. While Bendroflumethiazide and metformin are universally available, the findings reveal systemic weaknesses in respiratory medicine supply chains and geographical inequities, particularly for residents of Juaben as shown in S3 File (see Table 4). The percentages were calculated using the formula:

**Table 4:** Availability with emphasis on therapeutic groups across municipalities and facility type.

Therapeutic group and medicine availability	Municipality (n = 3)			Type of Facility (n = 3)		
	Oforikrom	Ejisu	Juaben	Government	Mission	Private
Cardiovascular diseases	67%	49%	39%	65%	47%	51%
Diabetes	72%	76%	52%	86%	67%	57%
Asthma	20%	53%	20%	40%	33%	27%
**Overall availability**	**67.2%**	**58.5%**	**54.6%**	**70.0%**	**63.2%**	**48.6%**

Note: Calculated as % of surveyed medicines present on the day of data collection (see supplementary information file 3).


$$\%=\frac{Number\;of\;facilities\;within\;each\;municipality\;with\;medicine\;present}{Number\;of\;facilities\;within\;the\;municipality}\;\times \;\text{1}\text{0}\text{0}$$

$$\%=\frac{Number\;of\;facilities\;within\;each\;facility\;group\;type\;with\;medicine\;present}{Number\;of\;facilities\;within\;the\;facility\;group\;type}\;\times \;\text{1}\text{0}\text{0}$$

### Association between medicine availability by municipality and facility type

The study identifies disparities in the availability of essential medicines across Ghana’s healthcare system, with both geographical and institutional inequities emerging. The chi-square test results reveal a distinct municipal gradient, with Oforikrom showing the highest medicine availability (70.0%), followed by Ejisu (63.2%) and Juaben, which trails significantly at 48.6% (p < 0.001). This 21.4 percentage point gap highlights municipal imbalances, particularly in Juaben, where medicines are unavailable for more than half the time, suggesting systemic supply chain deficiencies. Facility-type analysis shows government facilities maintain superior availability (67.2%) compared to mission (58.5%) and private (54.6%) providers (p = 0.042), likely due to their integration with government procurement systems (see Table 5).

**Table 5:** Association between medicine availability by municipality and facility type.

Variable	(N = 549)			p-value
Municipality	Oforikrom n (%)	Ejisu n (%)	Juaben n (%)	
Medicine availability				
Yes	132 (71.0%)	115 (63.2%)	88 (48.6%)	**<0.001**
No	54 (29.0%)	67 (36.8%)	93 (51.4%)	
**Facility type**	**Government n (%)**	**Mission n (%)**	**Private n (%)**	
Medicine availability				
Yes	123 (67.2%)	107 (58.5%)	100 (54.6%)	**0.042**
No	60 (32.8%)	76 (41.5%)	83 (45.4%)	

### Affordability of NCD medicines

This examination of medication affordability for patients across municipalities and facility types reveals disparities in treatment costs relative to the standard daily wage of GH₵ 14.88.

### Affordability analysis of selected NCD medicines for non-insured patients across municipalities

The study reveals substantial disparities in medication affordability across therapeutic categories and geographical locations. For hypertension treatments, Oforikrom emerges as the most expensive location for Bendroflumethiazide (0.71 days’ wages) and bisoprolol (1.77 days), while Juaben shows the highest costs for atenolol (1.05 days) and lisinopril (1.45 days). Diabetes medications present even greater financial burdens, with insulin premixed requiring 7.39–8.74 days’ wages across municipalities (the costliest treatment category). Striking geographical variations exist, exemplified by metformin’s twelve-fold price difference between Juaben (3.23 days) and Ejisu (0.26 days). Respiratory medications are available in limited supply, compounded by high costs, particularly for salbutamol inhalers in Oforikrom (4.07 days). Pain management medications demonstrate similar inequities, with tramadol costing 2.67 days’ wages in Oforikrom versus 1.31 in Juaben, and ibuprofen showing an eight-fold price variation across locations (see Fig 1).

### Affordability of selected NCD medicines across facility types for non-insured patients

The analysis reveals stark facility-level disparities in medication affordability, with private facilities imposing higher financial burdens across all therapeutic categories. For hypertension management, private facilities charge 2–3 times more than government facilities, with bisoprolol costing 2.18 days’ wages (vs 1.04 in government) and amlodipine costing 1.09 days’ wages (vs 0.40). Diabetes treatments show even more dramatic inequities: while insulin premixed costs 8.74 days’ wages in government/mission facilities, private facilities paradoxically offer it at 5.38 days. However, other diabetes medications, such as metformin (8.06 vs 1.05 days) and glimepiride (7.38 vs 1.57 days), remain substantially more expensive in private settings. Respiratory and cholesterol medications follow this pattern, with salbutamol inhalers (4.54 vs. 2.45 days) and atorvastatin (2.98 days in private) posing particular challenges. Even essential antibiotics show concerning disparities, with amoxicillin costing five times more in private facilities (1.24 vs. 0.25 days) (see Fig 2).

### Affordability of selected NCD medicines with added-on cost for insured patients across municipalities

This study highlights substantial geographical disparities in medication affordability across Ghanaian municipalities, with particularly pronounced variations for chronic disease treatments. Hypertension medications show distinct pricing patterns, ranging from relatively stable costs for Bendroflumethiazide (0.18–0.40 days’ wages) to fluctuations for amlodipine (0.20–0.36 days) and bisoprolol (unavailable in Ejisu but 1.17 days in Oforikrom). Diabetes treatments present even greater challenges, with insulin premixed being the most burdensome (3.94–5.44 days’ wages) and metformin showing a tenfold price difference between Juaben (1.61 days) and Oforikrom (0.16 days). While respiratory medicines like salbutamol inhalers remain limited to Ejisu (1.75 days), common analgesics and antibiotics are more affordable (paracetamol 0.18 days; amoxicillin 0.12–0.32 days) (see Fig 3).

### Affordability of selected NCD medicines for the added-on cost for insured patients across facility types

The study reveals alarming affordability challenges for essential medications across therapeutic categories, with insulin premixed emerging as the most financially burdensome treatment. Diabetic patients face substantial costs ranging from 3.94–5.44 days’ wages per vial across municipalities, with Ejisu (5.44 days) and Juaben (5.38 days) showing particularly high burdens. Other diabetes medications exhibit apparent municipal disparities: metformin costs vary tenfold between Oforikrom (0.16 days) and Juaben (1.61 days), while gliclazide remains unavailable in Oforikrom but costs 1.01 days’ wages in Juaben. Cardiovascular treatments also pose affordability concerns, particularly bisoprolol in Oforikrom (1.17 days) and atenolol/amlodipine approaching 0.5 days’ wages. Respiratory patients face access limitations, with salbutamol inhalers only available in Ejisu (1.75 days) (see Fig 4).

### Association between facility type and medicine affordability

The logistic regression analysis found an association between facility type and medicine affordability. Patients attending private health facilities were nearly four times more likely to face unaffordable medicines than those in government facilities (aOR = 3.85, 95% IC: 1.75–8.48) with a p-value of 0.001. Although patients in mission facilities also had higher odds of unaffordability (aOR = 1.24, 95% CI: 0.59–2.59), the association was not statistically significant (p = 0.572) (see Table 6).

**Table 6:** Logistic regression results on the association between facility type and medicine affordability.

Facility type	cOR (Exp[B])	95% CI	p-value	aOR (Exp[B])	95% CI	p-value
**Mission**	0.808	0.386 - 1.693	0.675	1.24	0.591 - 2.592	0.572
**Private**	0.260	0.118 - 0.572	**0.001**	3.85	1.747 - 8.483	**0.001**
**Government**	reference	–	–	reference	–	–

Table 7 outlines the main theme and sub-themes identified after the thematic analysis of the qualitative data.

**Table 7:** Availability and Affordability of Essential NCD Medicines: Theme and sub-themes.

Main theme	Sub-themes
Factors affecting the availability and affordability of NCD medicines	1.Availability of essential NCD medicines
	2.Affordability challenges for essential NCD medicines
	3.Suggestions for improvement

### Factors affecting the availability and affordability of NCD medicines in health facilities

1. **Availability of essential NCD medicines**

Our findings reveal systemic challenges in the availability of NCD medicines across primary healthcare facilities in the studied municipalities. The procurement lead time emerged as a critical factor, with government and mission facilities typically waiting two to three weeks for medicines from Regional Medical Stores, while private facilities often obtained supplies within one to two days. As one pharmacy manager noted,

*“It takes an average of two weeks to obtain medicines from the Regional Medical Stores.”* (P09)

Another respondent added,

*“The Central Medical Stores are sometimes not consistent with medicine supply. It can take two weeks or more.”* (P05)

In contrast, private facilities reported more efficient procurement:

*“We send the requisition list in the morning, and by the time the procurement person gets there later in the day, the medicines would be ready.”* (P07)

Three broad categories of factors affecting availability were identified: supplier-dependent, facility-dependent, and client-dependent factors. Supplier-related challenges included inconsistent delivery schedules and stockouts, as described by respondents:

*“Some suppliers do not honor delivery schedules. They deliver medicines in their own time.”* (P09)

Facility-level barriers included procurement delays and storage limitations:

*“Delays in the procurement processes... There is inadequate space in the pharmacy store.”* (P03), while client preferences for specific brands also influenced stock management

*“Some clients have their preferred brands... For example, Amlodipine.”* (P05)

1. **Affordability challenges for essential NCD medicines**

Affordability of NCD medicines was primarily influenced by procurement costs, which respondents attributed to exchange rate fluctuations, import taxes, and inflation. Additional cost drivers included transportation, facility overheads, and consumables required for treatment administration. One participant explained,

*“It depends on the cost at which we get the medicines... and also transportation cost”* (P02), while another noted,

*“Depending on the additional consumables... we add something small to cater for those”* (P04).

Facility-level financial constraints further exacerbated affordability issues, with participants citing a lack of sponsorship programs for NCD patients. Delays in reimbursement from the National Health Insurance Authority (NHIA) also strained resources:

*“NHIS doesn’t pay us early, and we don’t get enough to purchase some medicines on cash.”* (P04)

1. **Suggestions for improvement**

To enhance availability, respondents emphasized the need to expand local pharmaceutical production capacity, strengthen supplier accountability, and invest in infrastructure. As one participant stated,

*“The government can intervene to improve the capacity of local manufacturing companies.”* (P02), while another suggested,

*“Suppliers should be admonished to adhere strictly to delivery timelines.”* (P09).

Partnerships with NGOs were also proposed to supplement medicine supplies:

*“NGO programs... usually provide Amlodipine and Metformin.”* (P05).

For affordability, stakeholders recommended price regulation, donor support, and timely NHIA reimbursements.

*“There should be broader stakeholder engagements on price control.”* (P04)

One respondent urged, while others highlighted the need for:

*“Prompt reimbursement from NHIA to help procure medicines at good prices.”* (P08)

## Discussion

This study assessed access to medicine, operationally defined as availability and affordability, in three municipalities in Ghana. The findings from our comprehensive analysis of medicine availability and affordability across Ghanaian municipalities and healthcare facility types reveal systemic challenges that both align with and expand upon existing literature regarding access to essential medicines in LMICs. The disparities in therapeutic category availability, where diabetes medications were more accessible than cardiovascular drugs and particularly asthma treatments, mirror patterns observed in similar Sub-Saharan African contexts. A 2022 World Health Organization (WHO) report on NCD medicine availability noted comparable disparities across therapeutic categories, attributing them to vertical disease programs that prioritize certain conditions over others [1]. Our finding of asthma medication scarcity (particularly beclomethasone inhalers) aligns with multiple studies reporting that respiratory medicines are the most chronically undersupplied category across LMIC health systems [26,27], reflecting both cold chain challenges and market concentration among a few global manufacturers.

The persistent geographical disparities we observed, with Juaben consistently demonstrating the poorest availability of medicines across therapeutic groups, echo the “postcode lottery” phenomenon well documented in the global health literature. Our results substantiate Zameer et al.’s [28] findings in Pakistan, where peripheral districts showed 30–40% lower medicine availability compared to central regions, suggesting that supply chain weaknesses disproportionately affect certain geographical areas. The 28-percentage point gap between Oforikrom and Juaben in cardiovascular drug availability exceeds the 15–20% regional variation typically reported in similar studies [29], indicating particularly acute spatial inequities in Ghana’s pharmaceutical distribution system.

The superior availability of medicines in government facilities compared to private providers aligns with global evidence that government-sector procurement systems often achieve better medicine security. This supports Babar et al.’s [30] systematic review, which shows that government facilities in LMICs maintain 10–15% higher availability of essential medicines, attributed to bulk purchasing agreements and donor-supported supply mechanisms. However, our finding that this public-sector advantage narrows for asthma medications suggests condition-specific exceptions that merit further investigation, possibly related to different supply chains for respiratory products.

The affordability analysis revealed that even insured patients face substantial cost barriers, particularly for insulin and certain hypertension drugs. This challenges the assumption that health insurance automatically ensures access to medicine, supporting findings by Umar et al. [31] in Ghana’s NHIS system, where co-payments and medicine stock-outs undermined financial protection. The extreme variation in metformin prices exceeds the 2–3-fold price variations typically reported in LMIC medicine markets [32], suggesting local market distortions that require regulatory attention.

For non-insured patients, our findings showed that private facilities charge up to 8 days’ wages for metformin and 2 days’ wages for bisoprolol; this provides empirical support for Klazura et al.’s [33] systematic review of catastrophic health expenditure in LMICs. The finding that some medications cost more than a week’s wages aligns with recent Oxfam reports on medicine-induced poverty [34]. However, our identification of glimepiride’s 7–8 days’ wages in private facilities represents a newly quantified extreme in the literature.

The complete absence of critical medicines like atenolol 25 mg and beclomethasone inhalers across all locations corroborates Babar et al.’s [30] identification of an alarmingly low availability of beclomethasone in both government and private sectors across multiple LMICs, often resulting from tender design flaws and manufacturer discontinuations. Our data suggests these gaps may be more severe for certain cardiovascular and respiratory medications. These findings collectively underscore that achieving universal health coverage requires moving beyond insurance expansion to address systemic failures in the pharmaceutical market. The demonstrated need for geographically targeted interventions in underserved areas such as Juaben supports Bigdeli et al.’s [35] calls for precision public health approaches to improve access to medical care. The evidence suggests Ghana may benefit from: 1) therapeutic category-specific supply chain strengthening, particularly for asthma medications; 2) differential pricing regulations for private providers serving vulnerable populations; and 3) municipal-level medicine availability monitoring.

The observed challenges in the availability and affordability of NCD medicines mirror well-documented systemic barriers in LMICs. Like similar reports [1,35], our findings highlight how fragmented supply chains and delayed reimbursements disproportionately affect government facilities and rural areas, perpetuating treatment inequities. The two- to three-week procurement delays align with Wirtz et al.’s [29] reports of central medical store inefficiencies in Sub-Saharan Africa, while price volatility linked to import dependence corroborates evidence from Vialle-Valentin et al. [32]. These persistent gaps reinforce calls in recent literature [31,34] for integrated solutions: streamlined procurement to reduce lead times, mandatory NHIA reimbursement windows, and targeted subsidies for high-burden municipalities. As Juma et al. [35] emphasize, such localized, multi-sectoral approaches are critical to mitigate catastrophic expenditures and achieve universal NCD care coverage.

Our findings add to growing evidence that equitable access to essential medicines requires both health system strengthening and pharmaceutical market reforms tailored to local epidemiological and socioeconomic contexts.

### Strengths and limitations

A key strength of this study lies in its use of the WHO/HAI standardized methodology, which ensures comparability with international benchmarks while allowing for local adaptation. The integration of both quantitative and qualitative data provides a comprehensive understanding of medicine access challenges, capturing not only pricing and availability metrics but also contextual supply chain barriers. The inclusion of diverse facility types across three municipalities enhances the representativeness of the findings within the Ashanti Region. However, the study is limited by its cross-sectional design, which captures only a snapshot in time and may not reflect seasonal variations in medicine availability. Additionally, excluding tertiary facilities and cancer medicines narrows the scope for generalizability, and self-reported data from interviews may be subject to recall or social desirability bias. Notwithstanding these limitations, adherence to strict quality control measures in the study improved the validity, reliability, and policy utilities of our research findings.

## Conclusion and recommendations

This study highlights persistent inequities in the availability and affordability of essential NCD medicines in Ghana, with notable disparities across disease categories, facility types, and municipalities. Diabetes medicines were generally more available than cardiovascular and asthma treatments, while affordability remained a major concern, particularly in private facilities and underserved areas. These findings underscore that progress toward universal health coverage requires more than insurance expansion. Strengthening medicine supply chains, regulating private-sector pricing, and improving local production and monitoring mechanisms are essential to ensure equitable and sustainable access to NCD care in Ghana and similar low- and middle-income settings. Future research should investigate the drivers behind Juaben’s consistently poor performance and the unexpected glimepiride pricing anomaly in Ejisu. Longitudinal studies could assess whether observed patterns reflect chronic system failures or temporary stock fluctuations, and whether access variations translate into similar differences in treatment outcomes.

## Supporting information

S1 File Medicine median price ratios (MPRs) by municipality and facility type. (PDF)

S2 File 62 essential medicines for NCDs from the WHO PEN Tool list surveyed. (PDF)

S3 File Availability with emphasis on therapeutic groups across municipalities and facility types. (PDF)
