# Supplementary material for: Availability and affordability of essential medicines for non-communicable disease management in primary healthcare: Evidence from three municipalities in Ghana
Source: PLoS One. 2026 Apr 2;21(4):e0346140. doi: 10.1371/journal.pone.0346140 (PMC13046269; doi:10.1371/journal.pone.0346140)
Supplement: S1 File — (PDF) [file pone.0346140.s001.pdf]

Supplementary Information 1

Medicine median price ratios (MPRs) by municipality and facility type

Medicine Name	Municipality			Type of facility		
	Oforikrom	Ejisu	Juaben	Government	Mission	Private
1. Bendroflumethiazide tablet 2.5mg	2.75	1.34	1.10	0.86	1.34	2.75
2. Amlodipine tablet 10mg	0.45	0.74	0.48	0.45	0.74	0.90
3. Amlodipine tablet 5mg	0.79	1.66	2.27	0.66	1.74	1.98
4. Atenolol tablet 50mg	3.12	2.65	3.12	2.34	3.89	2.65
5. Atenolol tablet 25mg	0	0	0	0	0	0
6. Bisoprolol tablet 5mg	0.80	0.46	0.33	0.47	0.46	0.49
7. Propranolol tablet 40mg	1.81	0	0	0	2.31	0.66
8. Lisinopril tablet 10mg	0.40	0.31	0.68	0.20	0.49	0.52
9. Lisinopril tablet 5mg	0.87	0.90	1.26	0.82	0.60	0.91
10. Atorvastatin tablet 10mg	1.40	0.84	0.51	0.51	0	1.22
11. Atorvastatin tablet 20mg	1.12	0.42	0.52	0.41	0.26	0.58
12. Insulin Premixed (30/70) HM,100 units/ml in 10ml	3.69	4.13	2.68	3.89	4.36	1.34
13. Insulin Soluble HM 100units/ml in 10ml	1.75	1.41	1.82	1.82	1.57	1.32
14. Isophane Insulin Injection (HM), 100 units/ml in 10ml	0	0	4.06	4.06	0	0
15. Metformin tablet 500mg	1.03	0.64	2.06	0.68	1.03	3.09
16. Glibenclamide tablet 5mg	2.52	1.42	3.14	2.59	2.52	1.10
17. Gliclazide tablet 80mg	1.03	0.98	1.03	1.03	1.03	0.98
18. Glimepiride tablet 2mg	6.47	5.84	2.87	3.35	6.37	2.92
19. Isosorbide Dintitrate tablet 10mg	0.90	0	0	0.90	0	0
20. Glyceryl Trinitrate Sublingual tablet 500mcg	3.02	0	0	3.02	0	0
21. Furosemide tablet 40mg	10.08	3.23	4.03	3.23	12.63	5.04
22. Furosemide injection 10mg/ml in 2ml	3.65	6.29	2.52	2.52	6.29	2.33
23. Spirinolactone tablet 25mg	0.47	0	0	0	0.47	0
24. Spirinolactone tablet 50mg	1.95	1.59	0	1.59	0	1.59
25. Salbutamol Nebules 5mg	4.81	3.77	2.20	2.20	5.02	2.51
26. Salbutamol Nebules 2.5mg	3.98	7.32	2.74	2.74	4.57	3.66
27. Salbutamol Inhaler	1.40	2.04	0	1.40	2.04	1.15

28. Prednisolone tablet 5mg	4.95	1.39	0.53	0.53	3.12	0.75
29. Beclometasone inhaler, 100mcg/metered dose	0	0	0	0	0	0
30. Beclometasone inhaler, 200mcg/metered dose	0	0	0	0	0	0
31. Acetylsalicylic Acid tablet 75mg	1.30	1.69	1.52	1.69	1.58	1.18
32. Paracetamol tablet 500mg	3.02	2.87	5.17	2.87	2.87	4.02
33. Paracetamol Suppository 500mg	0.63	0.63	0.42	0.42	0.52	0.46
34. Ibuprofen 200mg tablet	2.21	4.04	1.23	2.45	3.00	2.21
35. Ibuprofen 400mg tablet	11.30	2.12	1.41	1.77	1.41	2.12
36. Codeine tablet 15mg	0	0	0	0	0	0
37. Codeine tablet 60mg	0	0	0	0	0	0
38. Tramadol capsule 50mg	5.17	3.12	3.61	3.18	3.90	3.12
39. Morphine Injection 10mg/ml	3.06	7.84	3.92	1.02	7.06	2.82
40. Morphine Sulphate tablet 10mg (slow release)	0	0	0	0	0	0
41. Morphine Sulphate tablet 30mg (slow release)	0	0	0	0	0	0
42. Erythromycin tablet 250mg	0.23	1.63	1.36	0	1.36	0.23
43. Amoxicillin capsules 500mg	1.91	0.82	1.61	0.63	1.41	1.81
44. Amoxicillin capsules 250mg	0.73	0.83	1.10	0.73	1.58	0.73
45. Hydrocortisone Succinate injection 100mg	1.84	1.92	0.80	1.25	3.21	1.76
46. Adrenaline injection 1mg/1ml (1:1000)	3.46	4.33	5.41	4.33	3.89	4.76
47. Enoxaparin Sodium Injection 40mg/0.4ml	0	0	0	0	0	0
48. Heparin injection 5000 iu/ml	0	0	0	0	0	0
49. Diazepam tablet 10mg	0.71	3.55	1.77	1.77	4.43	0.71
50. Diazepam tablet 5mg	0.18	1.23	0	0.42	1.39	0.18
51. Diazepam injection 5mg/ml in 2ml	0	0	0	0	0	0
52. Magnesium Sulphate injection 20% (10ml)	0	0	9.61	0	9.61	0
53. Magnesium Sulphate injection 50% (10ml)	2.21	0.98	1.09	1.81	1.59	1.27
54. Promethazine Hydrochloride injection 25mg/ml in 2ml	0.54	0.89	1.02	0.59	1.72	0.50
55. Promethazine Hydrochloride tablet 25mg	0	0	2.99	2.99	0	0
56. Promethazine Theoclate	0	0	0	0	0	0
57. Lactulose	1.66	2.68	1.82	1.66	22.48	0
58. Dextrose Infusion 10%	1.28	1.67	2.08	2.08	1.67	0.90
59. Dextrose Infusion 5%	1.32	1.53	2.08	1.67	1.71	1.25

60. Dextrose infusion 50%	0.53	0.28	0.55	0.46	0.64	0.21
61. Sodium chloride infusion 0.9% (500ml)	1.40	1.36	2.27	1.52	2.05	0.98
62. Oxygen	0	0	0	0	0	0

Note: MPR = Median local price/International Reference Price. WHO benchmarks for generics in LMICs: MPR ≤1.5 = affordable; >2 = overpriced
