# Supplementary material for: Availability and affordability of essential medicines for non-communicable disease management in primary healthcare: Evidence from three municipalities in Ghana
Source: PLoS One. 2026 Apr 2;21(4):e0346140. doi: 10.1371/journal.pone.0346140 (PMC13046269; doi:10.1371/journal.pone.0346140)
Supplement: S2 File — (PDF) [file pone.0346140.s002.pdf]

<u>**Supplementary Data 2**</u>

62 essential medicines for NCDs from the WHO PEN Tool list surveyed

Medicine Name
1. Bendroflumethiazide tablet 2.5mg
2. Amlodipine tablet 10mg
3. Amlodipine tablet 5mg
4. Atenolol tablet 50mg
5. Atenolol tablet 25mg
6. Bisoprolol tablet 5mg
7. Propranolol tablet 40mg
8. Lisinopril tablet 10mg
9. Lisinopril tablet 5mg
10. Atorvastatin tablet 10mg
11. Atorvastatin tablet 20mg
12. Insulin Premixed (30/70) HM,100 units/ml in 10ml
13. Insulin Soluble HM 100units/ml in 10ml
14. Isophane Insulin Injection (HM), 100 units/ml in 10ml
15. Metformin tablet 500mg
16. Glibenclamide tablet 5mg
17. Gliclazide tablet 80mg
18. Glimepiride tablet 2mg
19. Isosorbide Dintitrate tablet 10mg
20. Glyceryl Trinitrate Sublingual tablet 500mcg
21. Furosemide tablet 40mg
22. Furosemide injection 10mg/ml in 2ml
23. Spirinolactone tablet 25mg
24. Spirinolactone tablet 50mg
25. Salbutamol Nebules 5mg
26. Salbutamol Nebules 2.5mg
27. Salbutamol Inhaler
28. Prednisolone tablet 5mg
29. Beclometasone inhaler, 100mcg/metered dose
30. Beclometasone inhaler, 200mcg/metered dose
31. Acetylsalicylic Acid tablet 75mg
32. Paracetamol tablet 500mg
33. Paracetamol Suppository 500mg
34. Ibuprofen 200mg tablet
35. Ibuprofen 400mg tablet
36. Codeine tablet 15mg
37. Codeine tablet 60mg
38. Tramadol capsule 50mg
39. Morphine Injection 10mg/ml
40. Morphine Sulphate tablet 10mg (slow release)
41. Morphine Sulphate tablet 30mg (slow release)

42. Erythromycin tablet 250mg
43. Amoxicillin capsules 500mg
44. Amoxicillin capsules 250mg
45. Hydrocortisone Succinate injection 100mg
46. Adrenaline injection 1mg/1ml (1:1000)
47. Enoxaparin Sodium Injection 40mg/0.4ml
48. Heparin injection 5000 iu/ml
49. Diazepam tablet 10mg
50. Diazepam tablet 5mg
51. Diazepam injection 5mg/ml in 2ml
52. Magnesium Sulphate injection 20% (10ml)
53. Magnesium Sulphate injection 50% (10ml)
54. Promethazine Hydrochloride injection 25mg/ml in 2ml
55. Promethazine Hydrochloride tablet 25mg
56. Promethazine Theoclate
57. Lactulose
58. Dextrose Infusion 10%
59. Dextrose Infusion 5%
60. Dextrose infusion 50%
61. Sodium chloride infusion 0.9% (500ml)
62. Oxygen
