# Supplementary material for: Availability and affordability of essential medicines for non-communicable disease management in primary healthcare: Evidence from three municipalities in Ghana
Source: PLoS One. 2026 Apr 2;21(4):e0346140. doi: 10.1371/journal.pone.0346140 (PMC13046269; doi:10.1371/journal.pone.0346140)
Supplement: S3 File — (PDF) [file pone.0346140.s003.pdf]

Supplementary Information 3

Availability with emphasis on therapeutic groups across municipalities and facility types

Therapeutic Group & Medicine Name	Municipality (n=3)			Type of facility (n=3)		
	Oforikrom	Ejisu	Juaben	Government	Mission	Private
**Cardiovascular Diseases**						
Bendroflumethiazide tablet 2.5mg	100%	100%	100%	100%	100%	100%
Amlodipine tablet 10mg	100%	100%	67%	100%	100%	100%
Amlodipine tablet 5mg	100%	67%	100%	67%	100%	67%
Atenolol tablet 50mg	100%	100%	67%	100%	100%	67%
Atenolol tablet 25mg	0%	0%	0%	0%	0%	0%
Bisoprolol tablet 5mg	67%	33%	33%	67%	33%	33%
Propranolol tablet 40mg	67%	0%	0%	0%	33%	0%
Lisinopril tablet 10mg	67%	100%	100%	100%	100%	67%
Lisinopril tablet 5mg	67%	33%	67%	67%	67%	100%
Atorvastatin tablet 10mg	67%	67%	33%	100%	0%	67%
Atorvastatin tablet 20mg	67%	67%	33%	100%	67%	67%
Isosorbide Dintitrate tablet 10mg	33%	0%	0%	33%	0%	0%
Glyceryl Trinitrate Sublingual	33%	0%	0%	33%	0%	0%
Furosemide tablet 40mg	100%	33%	33%	100%	33%	33%
Spirinolactone tablet 25mg	0%	0%	0%	0%	0%	0%
Spirinolactone tablet 50mg	67%	33%	0%	33%	0%	67%
Acetylsalicylic Acid tablet 75mg	100%	100%	33%	100%	67%	100%
Group Average	***67%***	***49%***	***39%***	***65%***	***47%***	***51%***
**Diabetes**						
Insulin Premixed (30/70) HM, 100units, 10ml	67%	67%	33%	100%	33%	33%
Insulin Soluble HM, 100units/ml 10ml	67%	100%	33%	100%	33%	67%
Isophane Insulin Injection (HM), 100units, 10ml	0%	0%	33%	33%	0%	0%
Metformin tablet 500mg	100%	100%	100%	100%	100%	100%
Glibenclamide tablet 5mg	100%	67%	67%	67%	100%	67%
Gliclazide tablet 80mg	100%	100%	67%	100%	100%	67%

Glimepiride tablet 2mg	67%	100%	33%	100%	100%	67%
Group Average	***72%***	***76%***	***52%***	***86%***	***67%***	***57%***
**Asthma**						
Salbutamol Nebules 5mg	0%	67%	33%	33%	33%	33%
Salbutamol Nebules 2.5mg	67%	100%	67%	100%	100%	33%
Salbutamol Inhaler	33%	100%	0%	67%	33%	67%
Beclometasone dipropionate inhaler, 100mcg	0%	0%	0%	0%	0%	0%
Beclometasone dipropionate inhaler, 200mcg	0%	0%	0%	0%	0%	0%
Group Average	***20%***	***53%***	***20%***	***40%***	***33%***	***27%***
**Overall Availability**	**67.2%**	**58.5%**	**54.6%**	**70.0%**	**63.2%**	**48.6%**

NB: *Formula for municipalities: Number of facilities within each municipality with medicine present/Number of facilities within the municipality x 100*

NB: *Formula for facility type: Number of facilities within each facility group type with medicine present/Number of facilities within the facility group type x 100*
